# The association between toll-like receptor gene polymorphism and *Helicobacter pylori* infection risk: A systematic review and meta-analysis

**DOI:** 10.1097/MD.0000000000047788

**Published:** 2026-02-20

**Authors:** Zijie Xu, Xin Sun, Quanjiang Dong, Zihao Xu, Zhipeng Li, Lili Wang

**Affiliations:** a Department of Gastroenterology, Luoyang Central Hospital affiliated to Zhengzhou University, Luoyang, Henan, China; b Department of Gastrointestinal Endoscopy Center, Qingdao Municipal Hospital, Qingdao, Shandong, China; c Central Laboratories and Department of Gastroenterology, Qingdao Municipal Hospital, Qingdao, Shandong, China; d Department of Cardiology, Luoyang Central Hospital affiliated to Zhengzhou University, Luoyang, Henan, China; e Department of Gastroenterology, Yidu Central Hospital, Weifang, Shandong, China.

**Keywords:** *Helicobacter pylor*i, infection, meta-analysis, polymorphisms, susceptibility, toll-like receptors

## Abstract

**Background:**

Genetic polymorphisms in Toll-like receptor (*TLR*) genes have been implicated in host susceptibility to *Helicobacter pylori (H pylori*) infection. However, the extent to which specific single-nucleotide polymorphisms (SNPs) contribute to infection risk remains unclear. This study systematically evaluates the association between several *TLR* gene polymorphisms (*TLR1*, *TLR2*, *TLR4*, *TLR5*, *TLR9*, and *TLR10*) and susceptibility to *H pylori* infection, with the aim of identifying genetic loci with significant influence.

**Methods:**

A comprehensive literature search was conducted in PubMed, Embase, Web of Science, and China National Knowledge Infrastructure up to August 2024. Eligible studies were selected based on predefined inclusion criteria and assessed for methodological quality using the Newcastle–Ottawa scale. Pooled odds ratios (ORs) with 95% confidence intervals (CIs) were estimated using a random-effects model in Stata 17.0. Publication bias and sensitivity analyses were performed using SPSSAU.

**Results:**

A total of 22 studies comprising 11,610 participants (6052 experimental subjects and 5558 controls) were included, which examined a total of 10 SNPs of *TLR* genes. Notably, the *TLR*4 rs4986790 GG genotype (recessive model OR: 2.11; homozygous model OR: 1.78), the *TLR*4 rs4986791 TT genotype (recessive model OR: 4.11; homozygous model OR: 5.49), and the *TLR*10 rs10004195 AA genotype (recessive model OR: 1.64) were significantly associated with increased *H pylori* infection risk. Among these SNPs, *TLR*4 rs4986791 exhibited the strongest influence.

**Conclusion:**

The findings indicate that polymorphisms in *TLR*4 rs4986790, *TLR*4 rs4986791, and *TLR*10 rs10004195 significantly contribute to increased *H pylori* infection susceptibility. These genetic markers may facilitate risk stratification in healthy individuals and inform personalized health interventions based on genetic predisposition.

## 1. Introduction

*Helicobacter pylori (H pylori*) infection is one of the most widespread bacterial infections globally, affecting an estimated 4.4 billion individuals.^[1]^ The annual incidence of newly diagnosed *H pylori* infections has remained stable at approximately 10 million cases worldwide.^[2]^ *H pylori* infection is the primary risk factor for gastric cancer and low-grade mucosa-associated lymphoid tissue lymphoma, representing the first documented instance of a type of bacterial infection linked to carcinogenesis.^[3]^ Intriguingly, some individuals remain uninfected with *H pylori* throughout their lifetime.^[4]^ Host genetic predisposition and immune system health have been identified as critical determinants of susceptibility to *H pylori* infection.^[5,6]^

Recently, the toll-like receptor (*TLR*) family has garnered significant attention in the context of *H pylori* infection.^[7]^ TLRs, a class of host pattern recognition receptors, play a crucial role in pathogen detection, including recognition of *H pylori*.^[8]^ These receptors are essential for initiating the innate immune response.^[9]^ In humans, 10 TLRs have been identified in immune and gastric epithelial cells,^[10]^ with each recognizing distinct pathogen-associated molecular patterns. For example, TLR9 detects unmethylated cytosine-phosphate-guanine (CpG) motifs in microbial DNA, TLR5 recognizes flagellin, and TLR1, TLR2, TLR4, and TLR6 respond to bacterial lipopolysaccharides (LPS) and lipoproteins. Additionally, TLR2 detects peptidoglycan, while TLR3, TLR7, TLR8, TLR9, and TLR10 recognize microbial nucleic acids.^[11,12]^

Increasing evidence underscores the pivotal role of TLRs in modulating the host immune response to *H pylori* infection.^[13]^ Kareem et al utilized enzyme-linked immunosorbent assay techniques and observed elevated expression of TLR2 and TLR4 in *H pylori*-positive individuals compared to uninfected controls.^[14]^ Furthermore, gastric epithelial cells in children infected with *H pylori* exhibit upregulated expression of TLR2, TLR4, TLR5, and TLR9.^[15]^ In THP-1 monocytes, *H pylori* infection induces TLR8 expression and, to a lesser extent, TLR7 expression, with antagonism of TLR7/8 leading to a reduction in interferon-alpha (IFN-α) and interferon-beta (IFN-β) transactivation.^[16]^ Moreover, the *H pylori* Cag type IV secretion system (T4SS) translocates bacterial DNA into the host cell cytoplasm, stimulating intracellular TLR9 and initiating an anti-inflammatory signaling cascade.^[17]^ Collectively, these findings underscore the integral role of TLRs in host immune responses to *H pylori* infection.

Emerging evidence suggests that polymorphisms within TLR genes may serve as critical determinants of susceptibility to *H pylori* infection.^[18]^ Single-nucleotide polymorphisms (SNPs) within TLR genes may modulate receptor expression levels and disrupt downstream signaling pathways, thereby altering inflammatory mediator secretion and ultimately affecting host susceptibility to infection.^[19]^ There are numerous studies investigating the association between TLR gene polymorphisms and *H pylori* infection susceptibility, but findings remain inconsistent. The discrepancies may be attributed to differences in genetic backgrounds among different ethnic populations, as well as the limited statistical reliability of individual studies due to small sample sizes.

To address these inconsistencies, we conducted the first comprehensive systematic review and meta-analysis to quantitatively assess the association between TLR gene polymorphisms and *H pylori* infection susceptibility. By aggregating the available evidence, we aim to identify specific loci with significant influence to provide insight into the genetic predisposition to *H pylori* infection.

## 2. Materials and methods

The Preferred Reporting Items for Systematic Reviews and Meta-Analyses protocol for systematic review and meta-analysis was followed in conducting this meta-analysis.^[20]^ Scoping review following the guidelines of the Preferred Reporting Items for Systematic Reviews and Meta-Analyses Extension for Scoping Reviews. Ethical approval is not applicable for this analysis, which is based on publicly available data.

### 2.1. Literature search strategy

Two independent researchers systematically searched 4 major electronic databases – PubMed, EMBASE, Web of Science, and China National Knowledge Infrastructure – without language restrictions, encompassing literature from the inception of the databases to August 2024. To identify relevant studies on the association between *TLR* gene polymorphisms and *H pylori* infection susceptibility, the following search query was employed:

((((Toll-like receptors[Title/Abstract]) OR (Toll-like receptor[Title/Abstract])) OR (*TLR*[Title/Abstract])) AND (((((variant[Title/Abstract]) OR (polymorphism[Title/Abstract])) OR (mutation[Title/Abstract])) OR (genotype[Title/Abstract]))) AND (Helicobacter pylori infection[Title/Abstract]).

Additionally, a manual review of reference lists from relevant studies and systematic reviews was conducted to identify any additional publications meeting the inclusion criteria. The retrieved studies were subsequently screened based on predefined inclusion and exclusion criteria.

### 2.2. Inclusion and exclusion criteria

Studies were considered eligible for inclusion if they met the following criteria:

1. Study design: observational studies employing a case-control or cohort design investigating *TLR* gene polymorphisms as the exposure factor and *H pylori* infection risk as the outcome.
2. Data availability: studies providing explicit genotype frequency data, either directly reported or inferable from published results.
3. Hardy–Weinberg equilibrium (HWE): control group genotype distributions adhering to HWE.

Studies were excluded based on the following criteria:

1. Study type and data relevance: Absence of a control group, studies assessing gene expression rather than genetic polymorphisms, letters, conference abstracts, case reports, book sections, or studies lacking extractable genotype data.
2. Data completeness and redundancy: Studies with incomplete or duplicate datasets. In cases of multiple studies conducted by the same research group using overlapping populations, only the study with the largest sample size was included.
3. HWE violation: Studies in which the genotype distribution in the control group significantly deviated from HWE were excluded from the meta-analysis.

Two independent investigators screened the titles, abstracts, and full texts of the retrieved studies based on these criteria. Disagreements were resolved through discussion among the authors until a consensus was reached.

### 2.3. Data extraction and quality assessment

Two researchers independently extracted data using a standardized form. Any discrepancies were resolved through discussion, or by consulting the authors when necessary. The following key information was collected from each included study: author(s), publication year, country and ethnicity, sample sizes (cases and controls), genotype distribution and HWE conformity.

The methodological quality of the included studies was assessed using the Newcastle–Ottawa scale (NOS).^[21]^ The NOS evaluates studies based on 3 domains:

1. Selection of cases and controls (0–4 points).
2. Comparability between groups (0–2 points).
3. Assessment of exposure and outcome (0–3 points).

### 2.4. Statistical analysis

Genotype distribution data extracted from each study were analyzed using SPSSAU to calculate odds ratios (ORs) with 95% confidence intervals (CIs) for multiple genetic models. The association between *TLR* gene polymorphisms and *H pylori* infection susceptibility was assessed using pooled ORs with 95% CIs, employing a random-effects model. The following 5 genetic models were analyzed: the allelic, recessive, dominant, homozygote comparison, and heterozygote comparison models. Heterogeneity across studies was assessed using the *Q*-test and *I*^2^ statistics. A *P*-value < 0.10 or *I*^2^ > 50% was considered indicative of substantial heterogeneity.^[22]^ Subgroup analyses were conducted based on ethnicity to explore potential sources of heterogeneity.

Publication bias was assessed using funnel plots and Egger’s test, with statistical significance set at *P* < .05. Sensitivity analyses were also conducted to evaluate the robustness of the findings. All meta-analyses, heterogeneity assessments, and publication bias evaluations were performed using Stata version 17.0.

## 3. Results

### 3.1. Description of included studies and evaluation results

**Figure 1.**
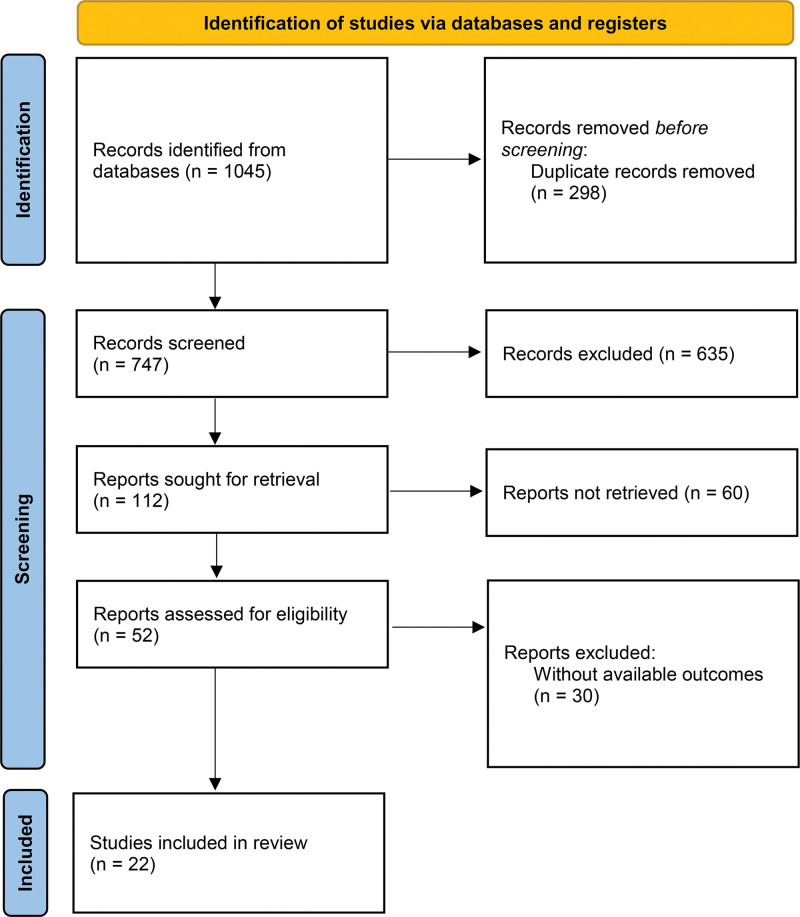
PRISMA-ScR flow diagram of the literature review process. PRISMA-ScR = Preferred Reporting Items for Systematic Reviews and Meta-Analyses Extension for Scoping Reviews.

A systematic search of electronic databases yielded 1045 publications. After removing duplicate records (n = 298) and excluding irrelevant studies based on title and abstract screening (n = 635), a total of 112 studies underwent full-text evaluation. Following the application of predefined inclusion and exclusion criteria, 90 studies were excluded. The selection process is illustrated in Figure 1, ultimately identifying 22 eligible publications for inclusion in the meta-analysis, encompassing a total sample size of 11,610 individuals (6052 experimental subjects and 5558 controls).^[23-44]^

These studies investigated the association between *H pylori* infection susceptibility and 10 SNPs within the *TLR* gene family, specifically: *TLR1* rs4833095, *TLR2* rs3804099, *TLR4* rs4986790, *TLR4* rs4986791, *TLR4* rs10759932, *TLR4* rs11536889, *TLR5* rs5744174, *TLR9* rs187084, *TLR9* rs352140, and *TLR10* rs10004195. The included studies were published between 2008 and 2024. Furthermore, an exhaustive review of literature assessing *TLR* gene SNPs involving fewer than 2 exploratory studies concluded that such variants were unsuitable for meta-analysis due to the limited availability of effect size estimates.

Among the included studies, 7 focused on Caucasian populations and 13 on Asian populations, while 1 study examined Romanians and another Latino population. Notably, no studies specifically targeted African populations. The genotype frequencies and study characteristics included in the meta-analysis are summarized in Table 1. Methodological quality was assessed using the NOS, with an average score of 8.05, indicating high methodological standards among the studies (Table 2).

**Table 1:** Characteristics of eligible studies considered for the association between toll-like receptor family gene polymorphism and *H pylori* infection risk in the meta-analysis.

Authors	Year	Country	Ethnicity	Sample size	HWE	Genotype frequency					
				HP (+)/HP (−)*		HP infection cases			Controls		
TLR1 rs4833095(C < T)			Minor allele C: 47.8%			TT	TC	CC	TT	TC	CC
Tang et al^[23]^	2015	China	Asian	1488/1018	Yes	199	660	629	140	499	379
Tas et al^[24]^	2020	Turkey	Caucasian	205/195	Yes	62	105	38	93	74	28
Tongtawee et al^[25]^	2018	Thailand	Asian	204/196	Yes	78	4	122	0	182	14
Dargiene et al^[26]^	2018	European countries	Caucasian	697/481	Yes	509	174	14	349	121	11
**TLR-2 rs3804099(C < T**)			**Minor allele C: 29.1%**			**TT**	**TC**	**CC**	**TT**	**TC**	**CC**
Mirkamandar et al^[27]^	2018	Iran	Caucasian	225/125	Yes	84	105	36	56	39	30
Tas et al^[24]^	2020	Turkey	Caucasian	205/195	Yes	78	99	28	102	75	18
Tongtawee et al^[28]^	2019	Thailand	Asian	204/196	Yes	125	28	51	131	41	24
Eed et al^[29]^	2020	Saudi Arabia	Caucasian	210/80	Yes	171	24	15	65	8	7
**TLR-4 rs4986790 (G < A**)			**Minor allele G: 14.7%**			**AA**	**AG**	**GG**	**AA**	**AG**	**GG**
Eed et al^[29]^	2020	Saudi Arabia	Caucasian	210/80	Yes	141	39	30	56	21	3
AL-Eitan et al^[30]^	2021	Jordan	Caucasian	223/217	Yes	219	4	0	209	8	0
He and Jiang^[31]^	2022	China	Asian	254/235	Yes	86	108	60	77	124	34
Loganathan et al^[32]^	2017	India	Asian	77/230	Yes	45	25	7	189	38	3
Tourani et al^[33]^	2018	Iran	Caucasian	56/44	Yes	50	6	0	41	3	0
Moura et al^[34]^	2008	Brazil	Latino	232/254	Yes	206	25	1	222	28	4
Meliţ et al^[35]^	2019	Romania	Romanian	50/97	Yes	48	2	0	92	5	0
Mirkamandar et al^[27]^	2018	Iran	Caucasian	225/125	Yes	180	33	12	95	25	5
Bagher et al^[36]^	2014	Iran	Caucasian	195/233	Yes	155	40	0	194	37	2
**TLR-4 rs4986791 (T < C**)			**Minor allele T: 14.9%**			**CC**	**CT**	**TT**	**CC**	**CT**	**TT**
Loganathan et al^[32]^	2017	India	Asian	77/230	Yes	38	30	9	202	22	6
Eed et al^[29]^	2020	Saudi Arabia	Caucasian	210/80	Yes	138	47	25	63	14	3
Tourani et al^[33]^	2018	Iran	Caucasian	56/44	Yes	49	5	2	42	2	0
Meliţ et al^[35]^	2019	Romania	Romanian	50/97	Yes	48	2		90	7	
**TLR-4 rs10759932 (C < T**)			**Minor allele C: 20.9%**			**TT**	**TC**	**CC**	**TT**	**TC**	**CC**
Tongtawee et al^[28]^	2019	Thailand	Asian	204/196	Yes	153	37	14	139	53	4
Jiang et al^[37]^	2018	China	Asian	236/242	Yes	145	80	11	128	89	25
**TLR-4 rs11536889 (G < C**)			**Minor allele G: 22.9%**			**CC**	**CG**	**GG**	**CC**	**CG**	**GG**
Tourani et al^[33]^	2018	Iran	Caucasian	56/44	Yes	0	19	37	0	18	26
AL-Eitan et al^[30]^	2021	Jordan	Caucasian	223/217	Yes	178	44	1	183	30	4
**TLR-5 rs5744174 (C < T**)			**Minor allele C: 20.4%**			**TT**	**TC**	**CC**	**TT**	**TC**	**CC**
Tas et al^[24]^	2020	Turkey	Caucasian	205/195	Yes	88	88	29	114	57	24
Goda et al^[38]^	2017	India	Asian	77/230	Yes	77	0	0	192	38	0
Zeng et al^[39]^	2011	China	Asian	382/362	Yes	243	139		225	137	
**TLR-9rs187084 (T < C**)			**Minor allele T: 45.4%**			**CC**	**CT**	**TT**	**CC**	**CT**	**TT**
Gao et al^[40]^	2020	China	Asian	121/151	Yes	10	70	41	33	66	52
Liang et al^[41]^	2024	China	Asian	240/390	Yes	144	64	32	126	166	98
**TLR-9rs352140 (T < C**)			**Minor allele T: 33.0%**			**CC**	**CT**	**TT**	**CC**	**CT**	**TT**
Loganathan et al^[32]^	2017	India	Asian	77/230	Yes	25	46	6	100	98	32
Eed et al^[29]^	2020	Saudi Arabia	Caucasian	210/80	Yes	106	81	23	39	35	6
**TLR-10 rs10004195 (A < T**)			**Minor allele A: 48.7%**			**TT**	**TA**	**AA**	**TT**	**TA**	**AA**
Eed et al^[29]^	2020	Saudi Arabia	Caucasian	210/80	Yes	25	61	124	17	30	33
Tang et al^[23]^	2015	China	Asian	1486/1008	Yes	276	712	498	207	493	308
AL-Eitan et al^[30]^	2021	Jordan	Caucasian	223/217	Yes	204	8	11	182	7	28
Tas et al^[24]^	2020	Turkey	Caucasian	205/195	Yes	114	37	54	150	38	7
Tongtawee et al^[25]^	2018	Thailand	Asian	204/196	Yes	135	10	59	51	123	22
Ram et al^[42]^	2015	Malaysia	Asian	57/28	Yes	15	18	24	8	11	9
Ying et al^[43]^	2016	China	Asian	418/234	Yes	98	190	130	37	125	72
Yu et al^[44]^	2014	China	Asian	201/182	Yes	60	87	54	56	93	33

Methods for confirming *H pylori* infection in the included literature: C13/14 breath test, rapid urease test, serum anti-*H pylori* antibody detection.

HP = *H pylori*, HWE = Hardy–Weinberg equilibrium.

**Table 2:** Quality assessment of the 8 case-control studies according to the Newcastle–Ottawa scale.

Authors	Selection of enrolled study subjects	Between-group comparability	Exposure outcomes and factors	Total
Tang^[23]^	4	2	2	8
Tas et al^[24]^	3	2	3	8
Tongtawee et al^[25]^	3	2	3	8
Dargiene et al^[26]^	3	2	2	7
Mirkamandar et al^[27]^	4	2	2	8
Tongtawee et al^[28]^	4	2	2	8
Eed et al^[29]^	4	2	2	8
AL-Eitan et al^[30]^	3	2	2	7
He and Jiang^[31]^	4	2	3	9
Loganathan et al^[32]^	4	2	3	9
Tourani et al^[33]^	3	2	3	8
Moura et al^[34]^	4	2	2	8
Meliţ et al^[35]^	3	2	2	7
Bagheri et al^[36]^	3	2	3	8
Jiang et al^[37]^	4	2	3	9
Goda et al^[38]^	4	2	2	8
Zeng et al^[39]^	3	2	2	7
Gao et al^[40]^	4	2	3	9
Liang et al^[41]^	4	2	2	8
Ram et al^[42]^	4	2	2	8
Ying et al^[43]^	4	2	2	8
Yu et al^[44]^	4	2	3	9
Average	3.64	2	2.41	8.05

### 3.2. Overall and ethnicity-based subgroup meta-analyses

Meta-analyses were conducted for the 10 identified polymorphic sites of 6 *TLR* genes. The overall and ethnicity-stratified meta-analysis results are summarized in Table 3.

**Table 3:** Main results and subgroup analysis by ethnicity for the meta-analysis of various types of toll-like receptors and *H pylori* infection susceptibility.

Type of toll-like receptors	Genetic model	Subgroups	Effect sizes	Test of association			Mode	Test of heterogeneity		Egger’s test of publication bias
				OR	95% CI	*P*		*I*^2^(%)	*P*	*P*
TLR1 rs4833095(C < T)	Allelic model	Overall	4	1.20	(1.01, 1.44)	.042	Random	62.80	.045	.497
		Asian	2	1.17	(1.01, 1.36)	.037	Random	24.70	.249	
		Caucasian	2	1.23	(0.76, 1.98)	.396	Random	85.10	.010	
	Recessive model	Overall	4	2.30	(0.71, 7.46)	.164	Random	96.00	<.001	.511
		Asian	2	4.81	(0.32, 71.24)	.254	Random	78.60	<.001	
		Caucasian	2	1.19	(0.76, 1.85)	.452	Random	0.00	.372	
	Dominant model	Overall	4	0.99	(0.55, 1.76)	.963	Random	88.50	<.001	.524
		Asian	2	12.81	(0.06, 2855.28)	.355	Random	93.30	<.001	
		Caucasian	2	0.71	(0.34, 1.50)	.371	Random	89.60	.002	
	CC vs TT	Overall	4	1.16	(0.66, 2.03)	.610	Random	63.90	.040	.554
		Asian	2	0.35	(0.02, 6.67)	.488	Random	77.30	.036	
		Caucasian	2	1.40	(0.61, 3.19)	.428	Random	64.30	.094	
	TC vs TT	Overall	3	1.20	(0.79, 1.82)	.386	Random	81.90	.004	.395
		Asian	1	0.93	(0.73, 1.19)	.568	–	–	–	
		Caucasian	2	1.42	(0.79, 1.82)	.362	Random	88.40	.003	
TLR-2 rs3804099(C < T)	Allelic model	Overall	4	1.26	(0.96, 1.66)	.089	Random	58.10	.067	.46
		Asian	3	1.16	(0.83, 1.61)	.393	Random	59.10	.087	
		Caucasian	1	1.59	(1.16, 2.18)	.004	–	–	–	
	Recessive model	Overall	4	1.18	(0.60, 2.35)	.629	Random	78.50	.003	.773
		Asian	3	0.91	(0.49, 1.70)	.765	Random	60.40	.080	
		Caucasian	1	2.39	(1.40 ,4.07)	.001	–	–	–	
	Dominant model	Overall	4	0.71	(0.57, 0.89)	.003	Fixed	0.00	.437	.256
TLR-2 rs3804099(C < T)	Dominant model	Asian	3	0.68	(0.52, 0.90)	.006	Fixed	17.30	.299	
		Caucasian	1	0.79	(0.52, 1.18)	.247	–	–	–	
	CC vs TT	Overall	4	1.36	(0.77, 2.40)	.283	Random	65.60	.033	.377
		Asian	3	1.12	(0.59, 2.13)	.726	Random	58.40	0.09	
		Caucasian	1	2.23	(1.29, 3.84)	.004	–	–	–	
	TC vs TT	Overall	4	1.30	(0.83, 2.03)	.257	Random	62.60	.046	.576
		Asian	3	**1.66**	**(1.23, 2.25**)	**.001**	Random	0.00	.644	
		Caucasian	1	0.72	(0.42, 1.23)	.225	–	–	–	
TLR-4 rs4986790 (G < A)	Allelic model	Overall	9	1.22	(0.89, 1.69)	.214	Random	66.00	.003	.773
		Caucasian	5	1.12	(0.84, 1.50)	.434	Random	16.00	.312	
		Asian	2	1.90	(0.71, 5.07)	.198	Random	92.40	<.001	
		Latino	1	0.81	(0.48, 1.36)	.423	–	–	–	
		Romanian	1	0.77	(0.15, 4.05)	.759	–	–	–	
	Recessive model	Overall	9	**1.92**	**(1.32, 2.78**)	**.001**	Fixed	27.80	.197	.58
		Caucasian	5	1.82	(0.86, 3.85)	.115	Fixed	4.20	.383	
		Asian	2	**2.11**	**(1.36, 3.28**)	**.001**	Random	72.70	.056	
		Latino	1	0.27	(0.03, 2.44)	.245	–	–	–	
		Romanian	1	1.93	(0.04, 98.43)	.743	–	–	–	
	Dominant model	Overall	9	0.88	(0.63, 1.24)	.470	Random	59.20	.012	.938
		Caucasian	5	0.97	(0.73, 1.29)	.841	Random	0.00	<.001	
		Asian	2	0.58	0.17, 1.94)	.373	Random	92.10	.431	
TLR-4 rs4986790 (G < A)	Dominant model	Latino	1	1.14	(0.66, 1.98)	.637	–	–	–	
		Romanian	1	1.30	(0.24, 6.97)	.756	–	–	–	
	GG vs AA	Overall	9	**1.78**	**(1.19, 2.65**)	**.005**	Fixed	37.00	.123	.729
		Caucasian	5	1.72	(0.81, 3.64)	.159	Fixed	0.00	.424	
		Asian	2	**1.98**	**(1.21, 3.22**)	**.006**	Fixed	37.00	.016	
		Latino	1	0.27	(0.03, 2.42)	.241	–	–	–	
		Romanian	1	1.91	(0.04, 97.95)	.748	–	–	–	
	GA vs AA	Overall	9	1.01	(0.72, 1.43)	.940	Random	56.40	.019	.991
		Caucasian	5	0.90	(0.62, 1.31)	.573	Random	26.80	.243	
		Asian	2	1.44	(0.42, 4.96)	.565	Random	91.50	.001	
		Latino	1	0.96	(0.54, 1.70)	.894	–	–	–	
		Romanian	1	0.77	(0.14, 4.11)	.757	–	–	–	
TLR-4 rs4986791 (T < C)	Allelic model	Overall	3	**3.38**	**(1.54, 7.41**)	**.002**	Random	73.30	.024	.896
		Asian	1	5.67	(3.48, 9.24)	<.001	–	–	–	
		Caucasian	2	**2.17**	**(1.32, 3.56**)	**.002**	Random	0.00	.701	
	Recessive model	Overall	3	**4.11**	**(1.88, 8.97**)	<**.001**	Fixed	0.00	.867	.328
		Asian	1	4.94	(1.70, 14.38)	.003	–	–	–	
		Caucasian	2	**3.32**	**(1.05, 10.45**)	**.04**	Fixed	0.00	0.844	
	Dominant model	Overall	4	0.41	(0.14, 1.17)	.097	Random	80.00	0.002	.529
		Asian	1	0.14	(0.07, 0.25)	<.001	–	–	–	
		Caucasian	2	0.50	(0.28, 0.88)	.017	Random	0.00	0.752	
TLR-4 rs4986791 (T < C)	Dominant model	Romanian	1	1.87	(0.37, 9.34)	.447	–	–	–	
	TT vs CC	Overall	3	**5.49**	**(2.49, 12.13**)	<**.001**	Fixed	0.00	.606	.497
		Asian	1	7.97	(2.68, 23.71)	<.001	–	–	–	
		Caucasian	2	**3.62**	**(1.14, 11.46**)	**.029**	Fixed	0.00	.825	
	CT vs CC	Overall	3	3.02	(0.92, 9.85)	.067	Random	81.70	.004	.881
		Asian	1	7.25	(3.78, 13.89)	<.001	–	–	–	
		Caucasian	2	1.60	(0.86, 2.98)	.136	Random	0.00	.718	
TLR-4 rs10759932 (C < T)	Allelic model	Overall	2	0.82	(0.55, 1.22)	.337	Random	63.70	.097	
	Recessive model	Overall	2	1.17	(0.15, 9.34)	.882	Random	89.50	.002	
	Dominant model	Overall	2	1.34	(1.01, 1.77)	.042	Random	0.00	.625	
	CC vs TT	Overall	2	1.06	(0.14, 8.33)	0954	Random	89.10	.002	
	CT vs TT	Overall	2	0.73	(0.54, 0.98)	.037	Random	0.00	.475	
TLR-4 rs11536889 (G < C)	Allelic model	Overall	2	1.22	(0.83, 1.78)	.317	Fixed	0.00	.908	
	Recessive model	Overall	2	0.78	(0.16, 3.76)	.754	Random	52.20	.148	
	Dominant model	Overall	2	0.74	(0.45, 1.20)	.217	Fixed	0.00	.973	
	GG vs CC	Overall	2	0.39	(0.06, 2.65)	.333	Fixed	0.00	.461	
	CG vs CC	Overall	2	1.50	(0.91, 2.48)	.115	Fixed	0.00	.861	
TLR-5 rs5744174 (C < T)	Allelic model	Overall	2	0.31	(0.01, 11.40)	.522	Random	84.80	.01	
	Recessive model	Overall	2	1.20	((0.67, 2.13)	.538	Fixed	0.00	.646	
	Dominant model	Overall	3	1.01	(0.43, 2.35)	.982	Random	85.50	.001	.667
		Caucasian	1	0.53	(0.36, 0.79)	.002	–	–	–	
TLR-5 rs5744174 (C < T)	Dominant model	Asian	2	4.26	(0.16, 109.97)	.383	Random	81.80	.019	
	CC vs TT	Overall	2	1.58	(0.87, 2.89)	.135	Fixed	0.00	.82	
	TC vs TT	Overall	2	0.32	(0.01, 17.99)	.580	Random	87.80	.004	
TLR-9rs187084 (T < C)	Allelic model	Overall	2	0.70	(0.25, 1.90)	.480	Random	95.50	<.001	
	Recessive model	Overall	2	0.66	(0.32, 1.39)	.274	Random	79.70	.026	
	Dominant model	Overall	2	1.03	(0.11, 9.62)	.978	Random	96.60	<.001	
	TT vs CC	Overall	2	0.84	(0.10, 7.31)	.875	Random	95.30	<.001	
	CT vs CC	Overall	2	1.06	(0.11, 10.48)	.962	Random	96.40	<.001	
TLR-9rs352140 (T < C)	Allelic model	Overall	2	1.08	(0.82, 1.42)	.592	Fixed	0.00	.819	
	Recessive model	Overall	2	0.89	(0.31, 2.51)	.820	Random	60.70	.111	
	Dominant model	Overall	2	0.82	(0.49, 1.40)	.472	Random	49.70	.159	
	TT vs CC	Overall	2	1.03	(0.52, 2.05)	.933	Fixed	0.00	.369	
	CT vs CC	Overall	2	1.26	(0.58, 2.74)	.260	Random	74.80	.046	
TLR-10 rs10004195 (A < T)	Allelic model	Overall	8	1.21	(0.79, 1.59)	.512	Random	91.90	<.001	.883
		Caucasian	3	1.42	(0.44, 4.57)	.555	Random	96.20	<.001	
		Asian	5	0.96	(0.76, 1.22)	.729	Random	77.20	.002	
	Recessive model	Overall	8	**1.64**	**(1.04, 2.58**)	**.034**	Random	87.20	<.001	.308
		Caucasian	3	1.89	(0.36, 10.00)	.455	Random	94.50	<.001	
		Asian	5	**1.49**	**(1.03, 2.15**)	**.033**	Random	74.60	.003	
	Dominant model	Overall	8	1.14	(0.65, 2.03)	.646	Random	92.70	<.001	.737
		Caucasian	3	0.72	(0.25, 2.09)	.551	Random	90.60	<.001	
TLR-10 rs10004195 (A < T)	Dominant model	Asian	5	1.50	(0.74, 3.05)	.264	Random	93.40	<.001	
	AA vs TT	Overall	8	1.37	(0.82, 2.29)	.235	Random	85.30	<.001	.623
		Caucasian	3	2.07	(0.31, 13.71)	.451	Random	94.70	<.001	
		Asian	5	1.09	(0.84, 1.43)	.514	Random	34.20	.194	
	TA vs TT	Overall	8	0.64	(0.32, 1.25)	.191	Random	92.50	<.001	.386
		Caucasian	3	1.27	(0.85, 1.87)	.240	Random	0.00	.895	
		Asian	5	0.44	(0.16, 1.77)	.100	Random	95.50	<.001	

Results with significant correlation (*P* < .05) are emphasized by the bold values.

CI = confidence interval, OR = odds ratio, *P* = *P*-value.

#### 3.2.1. TLR4 rs4986790 and H pylori susceptibility

**Figure 2.**
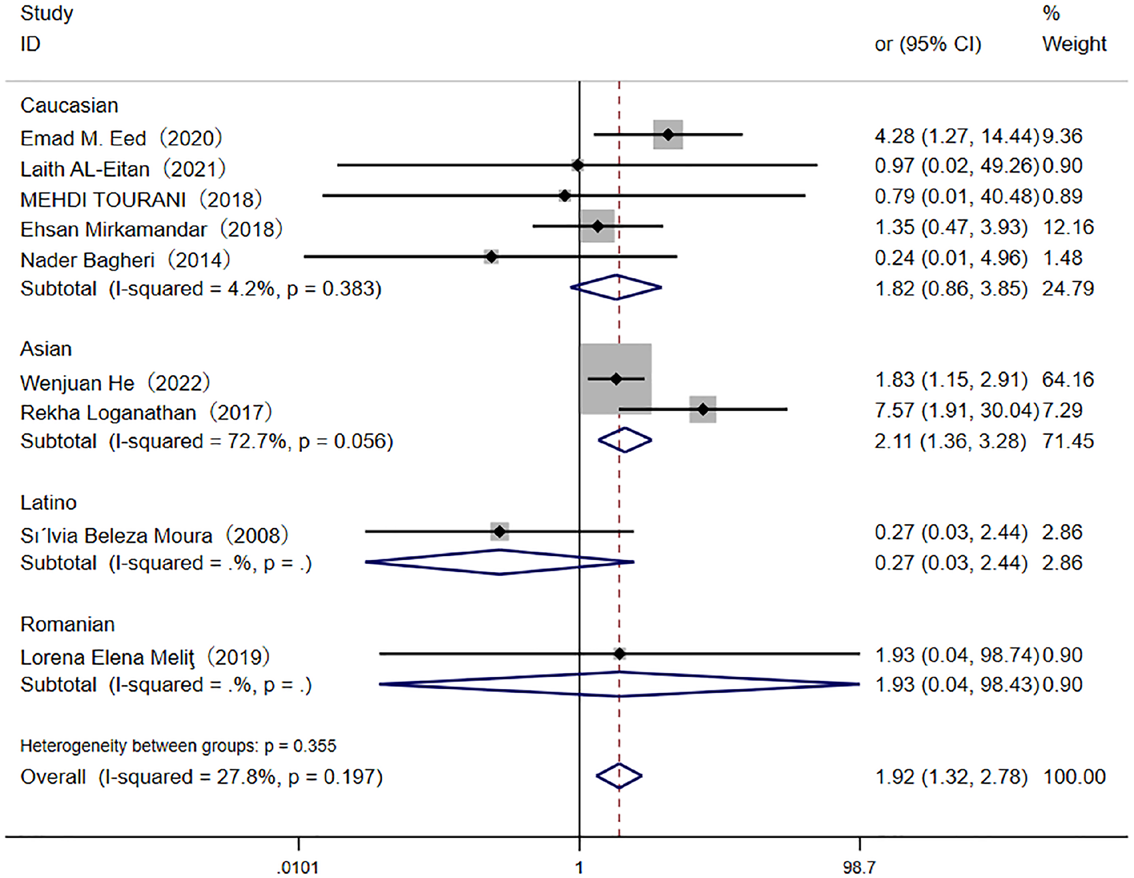
Forest plot for the association between *TLR-4* rs4986790 (G < A) polymorphism and *H pylori* infection risk in recessive model. CI = confidence interval.

**Figure 3.**
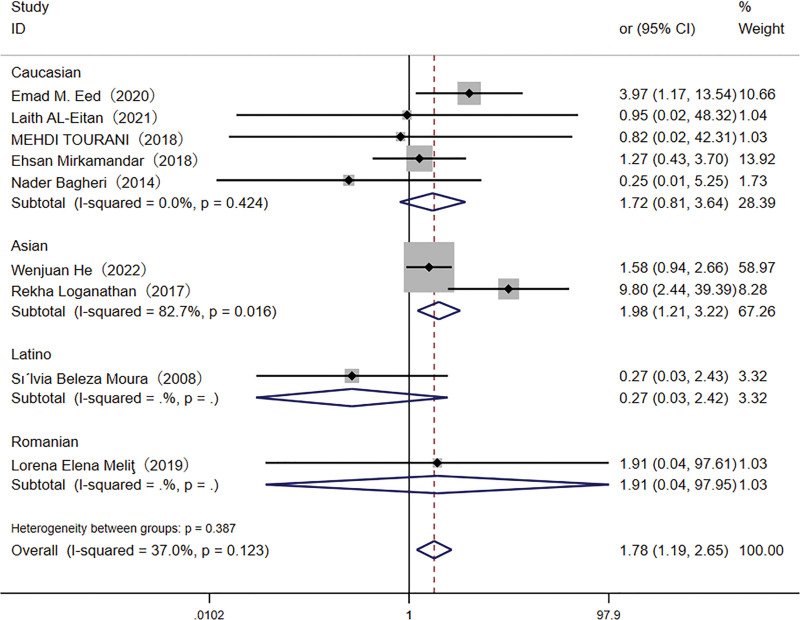
Forest plot for the association between *TLR-4* rs4986790 (G < A) polymorphism and *H pylori* infection risk in homozygote (GG vs AA) model. CI = confidence interval.

A total of 3037 participants were included in the meta-analysis assessing the association between the *TLR4* rs4986790 polymorphism and *H pylori* infection susceptibility. The GG genotype was present in 7.2% of infected individuals compared to 3.4% of controls. Pooled analysis demonstrated a significant association between the GG genotype and an increased risk of *H pylori* infection, as evidenced by both the recessive model (OR: 1.92, 95% CI: 1.32–2.78) and the homozygote model (OR: 1.78, 95% CI: 1.19–2.65; Figs. 2 and 3). In the Asian subgroup, the TT genotype was associated with a higher risk of *H pylori* infection in the recessive (OR: 2.11, 95% CI: 1.36–3.28) and homozygote (OR: 1.98, 95% CI: 1.21–3.22) models.

#### 3.2.2. TLR4 rs4986791 and H pylori susceptibility

**Figure 4.**
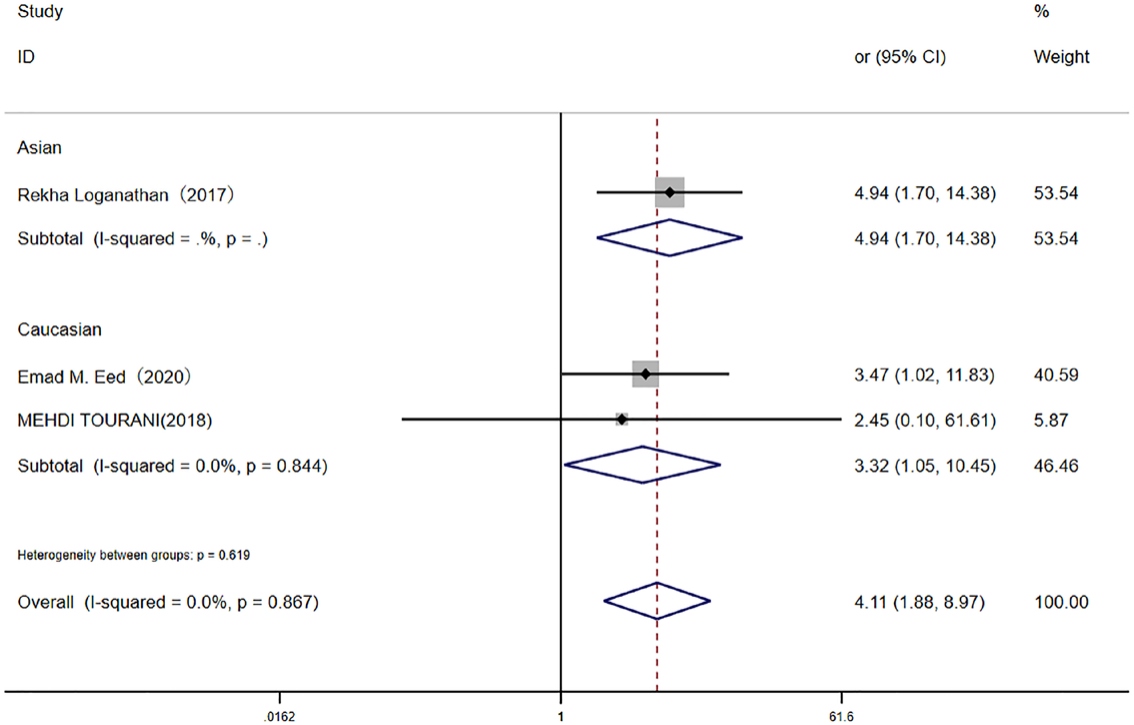
Forest plot for the association between *TLR-4* rs4986791 (T < C) polymorphism and *H pylori* infection risk in recessive models. CI = confidence interval.

**Figure 5.**
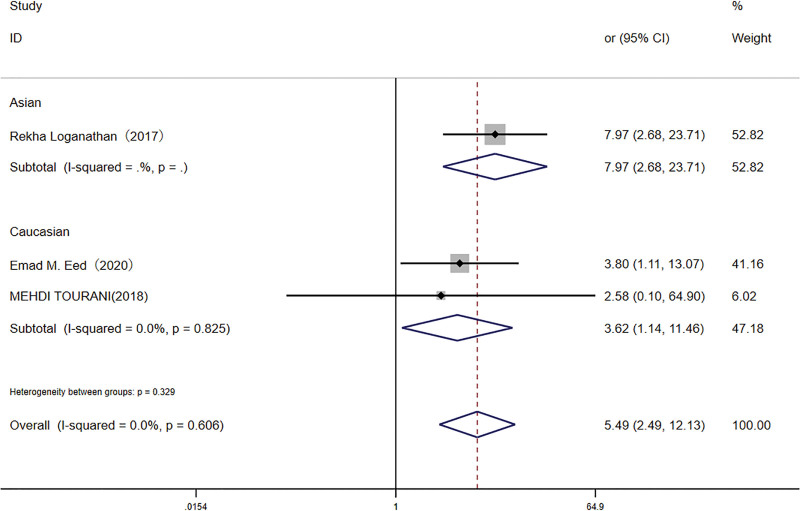
Forest plot for the association between *TLR-4* rs4986791 (T < C) polymorphism and *H pylori* infection risk in homozygote (TT vs CC) model. CI = confidence interval.

An additional meta-analysis, including 843 participants, examined the *TLR4* rs4986791 polymorphism. The TT genotype was detected in 10.2% of infected individuals and 2.5% of controls. The presence of the TT genotype was significantly associated with increased *H pylori* infection risk in both the recessive model (OR: 4.11, 95% CI: 1.88–8.97) and the homozygote model (OR: 5.49, 95% CI: 2.49–12.13; Figs. 4 and 5) Subgroup analysis in the Caucasian population demonstrated a similar association, albeit with a smaller effect size.

#### 3.2.3. TLR10 rs10004195 and H pylori susceptibility

**Figure 6.**
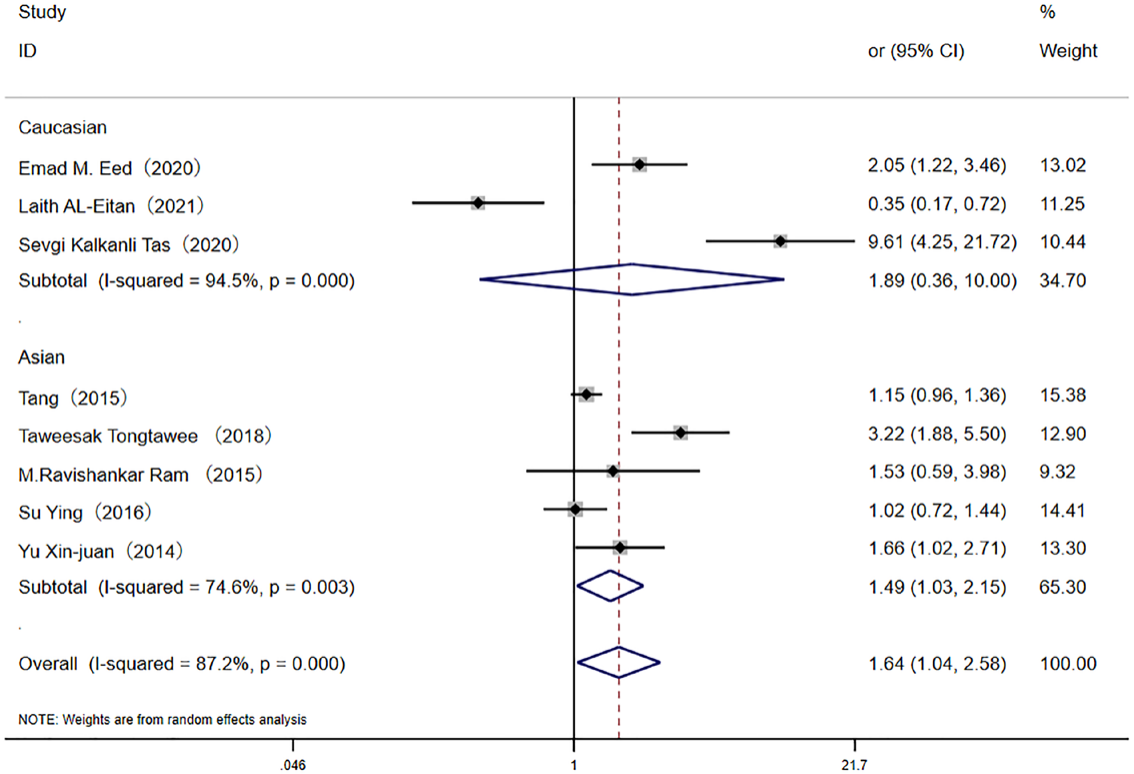
Forest plot for the association between *TLR-10* rs10004195 (A < T) polymorphism and *H pylori* infection risk in recessive model. CI = confidence interval.

The association between *TLR10* rs10004195 and *H pylori* susceptibility was assessed in 5144 participants, with the AA genotype observed in 31.8% of infected individuals and 23.9% of controls. The recessive model revealed a significant association between the AA genotype and increased infection risk (OR: 1.64, 95% CI: 1.03–2.58). In the Asian subpopulation, this association was further confirmed (OR: 1.49, 95% CI: 1.03–2.15), as depicted in Figure 6.

#### 3.2.4. Other polymorphisms and H pylori susceptibility

*TLR1* rs4833095 exhibited a weak association with *H pylori* infection, reaching statistical significance only in the allelic model (OR: 1.20, 95% CI: 1.01–1.44). This association was confirmed within the Asian subgroup (allelic OR: 1.17, 95% CI: 1.01–1.39), though the effect size was modest.

*TLR4* rs10759932 showed a potential association with increased susceptibility to *H pylori* infection in the dominant model (OR: 1.34, 95% CI: 1.01–1.77); however, further validation is required due to the limited number of studies available.

Unlike the overall meta-analysis findings, an association between *TLR2* rs3804099 and *H pylori* infection was only observed in the Asian subgroup in the heterozygote (CT vs TT) model (OR: 1.66, 95% CI: 1.23–2.25).

## 4. Summary of findings

In conclusion, our meta-analysis provides robust evidence that the *TLR4* rs4986790, *TLR4* rs4986791, and *TLR10* rs10004195 polymorphisms are significantly associated with increased susceptibility to *H pylori* infection. These findings contribute to a deeper understanding of host genetic factors influencing *H pylori* infection risk and may have implications for future risk stratification and targeted interventions.

### 4.1. Systematic review of less-studied polymorphisms in TLR genes and *H pylori* infection

Cabrera-Andrade et al^[45]^ explored the association between *TLR* gene polymorphisms (*TLR1* 1805T/G, *TLR2* 2029C/T, *TLR4* 896A/G) and susceptibility to *H pylori* infection in an Ecuadorian cohort. Their findings indicated that only the *TLR1* 1805G allele exhibited a protective effect against *H pylori* infection, while no significant associations were observed in the other polymorphisms. In a study conducted in China, Zhao et al^[46]^ examined multiple SNPs (*TLR2*: rs3804100, rs7696323, rs10116253; *TLR4*: rs10983755, rs11536878, rs1927914, rs7873784) but did not identify any significant correlations between specific SNPs and *H pylori* infection. In 2020, Gao et al reported an inverse association between *TLR9* rs164640 AA homozygosity and the likelihood of *H pylori* infection in a Chinese population.^[40]^ Based on the current limited data, these *TLR* SNPs have not been conclusively linked to increased *H pylori* infection risk, underscoring the necessity for further research to elucidate their potential role in host susceptibility.

### 4.2. Inter-study publication bias and sensitivity analysis

A publication bias analysis was performed for each *TLR* gene polymorphism included in this study. Egger’s test revealed no significant evidence of publication bias for the majority of SNP analyses (*P* = .256–0.991), with funnel plots presented in the Figures S1–S6, Supplemental Digital Content, https://links.lww.com/MD/R430. Sensitivity analyses confirmed the robustness of the findings, as the pooled effect sizes remained stable despite the exclusion of any given study.

## 5. Discussion

This study represents the first comprehensive systematic review and meta-analysis investigating the association between all prevalent SNPs within the *TLR* gene family and *H pylori* infection susceptibility. Our findings provide robust evidence that the recessive homozygous genotypes of *TLR4* rs4986790, *TLR4* rs4986791, and *TLR10* rs10004195 are significantly associated with an increased risk of *H pylori* infection.

The close relationship between *H pylori* infection and host immune responses has been well established.^[9]^ TLRs specifically recognize *H pylori* antigens and activate both specific and nonspecific immune responses.^[11]^ Polymorphisms within the *TLR* gene family may impair receptor function, leading to altered immune signaling and affecting host susceptibility to *H pylori* infection.^[47]^

Previous meta-analyses have primarily focused on the *TLR4* rs4986790 variant. He and Jiang conducted a meta-analysis of 7 studies and identified a significant association between *TLR4* rs4986790 and *H pylori* infection risk in Asian populations.^[31]^ By incorporating additional studies, our analysis confirmed this association in both the overall population and Asian subgroup. This study also presents the first meta-analysis demonstrating a significant association between *TLR4* rs4986791 and *H pylori* infection susceptibility. Prior research has linked *TLR4* rs4986791 to other infectious diseases, reinforcing the relevance of the gene in host-pathogen interactions. For example, Sljivancanin Jakovljevic et al reported an association between *TLR4* rs4986791 and an increased risk of neonatal culture-proven sepsis,^[48]^ while Schurz et al found that the presence of the T allele at this locus was associated with a higher risk of tuberculosis in the Asian population.^[49]^ These findings suggest that *TLR4* rs4986791 is a key variant influencing genetic susceptibility to infections, including *H pylori*. The *TLR10* rs10004195 polymorphism has been previously investigated with inconsistent results regarding *H pylori* susceptibility. This study, as the first pooled analysis, provides conclusive evidence that individuals carrying the AA genotype have a significantly increased risk of *H pylori* infection.

### 5.1. TLR signaling and its role in *H pylori* infection

TLR signaling plays a critical role in shaping the immune microenvironment during pathogenic infections. Variations in *TLR* genes can disrupt signaling cascades, alter ligand binding affinities, and modulate immune responses to pathogens such as *H pylori*, leading to dysregulated inflammatory responses.^[50]^ These polymorphisms may also affect the recruitment and activation of immune cells and influence *H pylori* immune evasion activity, thereby impacting host susceptibility to *H pylori* infection.^[51]^

TLR4 contains an extracellular domain that initiates a signaling cascade upon binding to *H pylori* LPS. This cascade leads to the release of cytokines such as IL-1, NF-κB, IRF-3, and TNF-α, as well as the activation of immune-related genes through both MyD88-dependent and MyD88-independent pathways, ultimately generating an immune response targeting *H pylori* infection.^[52]^ A review of the NCBI database revealed that *TLR4* rs4986790 and *TLR4* rs4986791 are missense mutations. Specifically, rs4986790 involves an A-to-G substitution, resulting in an amino acid change from aspartic acid to glycine, while rs4986791 features a C-to-T transition, converting threonine to isoleucine. Notably, these 2 SNP sites do not exhibit linkage disequilibrium. A study by Hold et al compared *TLR4* variants rs4986790 and rs4986791 to wild-type *TLR4* (WT-*TLR4*) in terms of LPS-induced reactivity. In HEK cells, the rs4986791 variant led to constitutive NF-κB activation while exhibiting a low response to LPS stimulation compared to WT-*TLR4*. Furthermore, monocytes and macrophages from carriers of these polymorphisms demonstrated a 0.6-fold decrease in NF-κB activation and a 12-fold increase in IFN-β expression upon LPS stimulation compared to wild-type cells. These functional changes were associated with significant alterations in cytokine profiles, leading the study to conclude that *TLR4* SNPs modulate receptor activity, thereby influencing the host’s immune response to LPS and resulting in a dysregulated immune response to infection.^[53]^

TLR10 functions as a pattern recognition receptor that forms a heterodimer with TLR2, facilitating the recognition of *H pylori* LPS.^[54-56]^ Gastric biopsies from *H pylori*-infected individuals reveal increased *TLR*10 mRNA and protein expression in gastric epithelial cells. Exposure to heat-killed *H pylori* or its LPS activates NF-κB via the TLR2/10 heterodimer, with significant involvement of the TLR2 subfamily.^[57]^ Additionally, another study demonstrated that TLR10 and its heterodimer on gastric mucosal epithelium bolster the immune response to *H pylori* infection by amplifying NF-κB activation and interleukin-1β secretion.^[58]^ These findings underscore the pivotal role of TLR10 in mediating immune responses to *H pylori* infection. Variations in the *TLR10* gene may disrupt the balance between pro-inflammatory and anti-inflammatory responses, thereby influencing susceptibility to infection.^[59]^ Specifically, the SNP rs10004195, located in the TLR*10* promoter region, is hypothesized to reduce the ability of the TLR2/10 heterodimer to recognize *H pylori* LPS, consequently impairing immune responses.^[60]^ Furthermore, the AA homozygous genotype at *TLR10* rs10004195 has been associated with increased inflammation in Thai patients with *H pylori*-induced gastritis.^[56]^ Collectively, these findings suggest that *TLR10* rs10004195 plays a role in *H pylori* recognition and regulates TLR2/10 heterodimer function, thereby regulating immune responses to infection.

### 5.3. Study contribution and meta-analysis findings

Our systematic review and meta-analysis constitute the first large-scale study to comprehensively assess the relationship between *TLR* gene family SNPs and *H pylori* infection susceptibility. This study also provides the first meta-analysis demonstrating sufficient evidence for a significant association between *TLR*4 rs4986791 and *TLR*10 rs10004195, both of which are linked to increased susceptibility to *H pylori* infection. Additionally, our study is pioneering in its exploration of the association between *H pylori* infection susceptibility and SNPs in *TLR1* (rs4833095), *TLR*2 (rs3804099), *TLR*4 (rs10759932, rs11536889), *TLR*5 (rs5744174), and *TLR*9 (rs187084, rs352140). Importantly, our analysis detected no significant publication bias, and studies with genotype distributions deviating from HWE were excluded from further analysis to ensure data integrity.

### 5.4. Study limitations and future directions

Despite the comprehensive nature of this meta-analysis, several limitations must be acknowledged. First, significant heterogeneity was observed among the included studies. To mitigate this and improve the reliability of the results, a random-effects model was employed for statistical analysis. Stratified analysis revealed that ethnicity was a major contributor to heterogeneity for several polymorphic loci, highlighting the necessity of considering this factor in future investigations of *TLR* polymorphism and *H pylori* infection risk.

Second, the interaction between genetic and environmental factors – such as diet, alcohol consumption, smoking, and physical activity – may influence *H pylori* infection susceptibility. However, due to data constraints, interaction analysis was not feasible in the present study. Future research should integrate genetic and environmental data to provide a more comprehensive understanding of *H pylori* infection risk.

Third, research on certain SNPs remains limited (e.g., TLR-4 rs10759932, *TLR*5 rs5744174, *TLR*9 rs352140), leading to insufficient evidence to confirm their association with *H pylori* infection susceptibility.^[28,29,37-39]^ Additional large-scale, multicenter studies are necessary to validate these findings and investigate potential novel polymorphisms in the *TLR* gene family.

Furthermore, the status of virulence factors (e.g., CagA, VacA) were not mentioned in the included studies. Therefore, a stratified analysis based on *H pylori* virulence factor status could not be performed.^[61]^ Future studies should further explore the association between *H pylori* virulence factor status to host *TLR* gene polymorphisms.

Lastly, the lack of data on African populations restricts the generalizability of our findings. Given that genetic variations in immune response genes exhibit population-specific patterns, further studies are required to assess the impact of *TLR* polymorphisms in African cohorts, thereby increasing the global applicability of these findings.

## 6. Conclusion

In summary, this meta-analysis provides compelling evidence for a significant association between *TLR*4 rs4986790, *TLR*4 rs4986791, and *TLR*10 rs10004195 polymorphism and increased susceptibility to *H pylori* infection. These findings have important implications for clinical practice, as identifying individuals carrying these polymorphisms could facilitate early detection and targeted prevention strategies, potentially reducing the burden of *H pylori*-related diseases, including gastric cancer and peptic ulcer disease.

Future research should prioritize well-designed, large-scale studies encompassing diverse ethnic populations, particularly African cohorts, to address the current gap in genetic epidemiology. Additionally, further investigations into less-studied SNPs and their potential functional effects are warranted. Studies integrating gene–gene and gene–environment interactions will be crucial in advancing our understanding of TLR polymorphisms and their role in *H pylori* infection susceptibility, ultimately contributing to the development of personalized medical interventions.

## Author contributions

**Conceptualization:** Zijie Xu, Quanjiang Dong.

**Data curation:** Zijie Xu.

**Formal analysis:** Quanjiang Dong, Zihao Xu.

**Funding acquisition:** Quanjiang Dong.

**Investigation:** Lili Wang.

**Methodology:** Zijie Xu, Zhipeng Li, Lili Wang.

**Project administration:** Xin Sun.

**Resources:** Xin Sun.

**Software:** Zihao Xu, Zhipeng Li.

**Supervision:** Zijie Xu, Quanjiang Dong.

**Validation:** Zhipeng Li, Lili Wang.

**Visualization:** Lili Wang.

## Supplementary Material
