# Supplementary material for: The association between toll-like receptor gene polymorphism and Helicobacter pylori infection risk: A systematic review and meta-analysis
Source: Medicine (Baltimore). 2026 Feb 20;105(8):e47788. doi: 10.1097/MD.0000000000047788 (PMC12928952; doi:10.1097/MD.0000000000047788)
Supplement: Supplementary file 1 [file medi-105-e47788-s001.pdf]

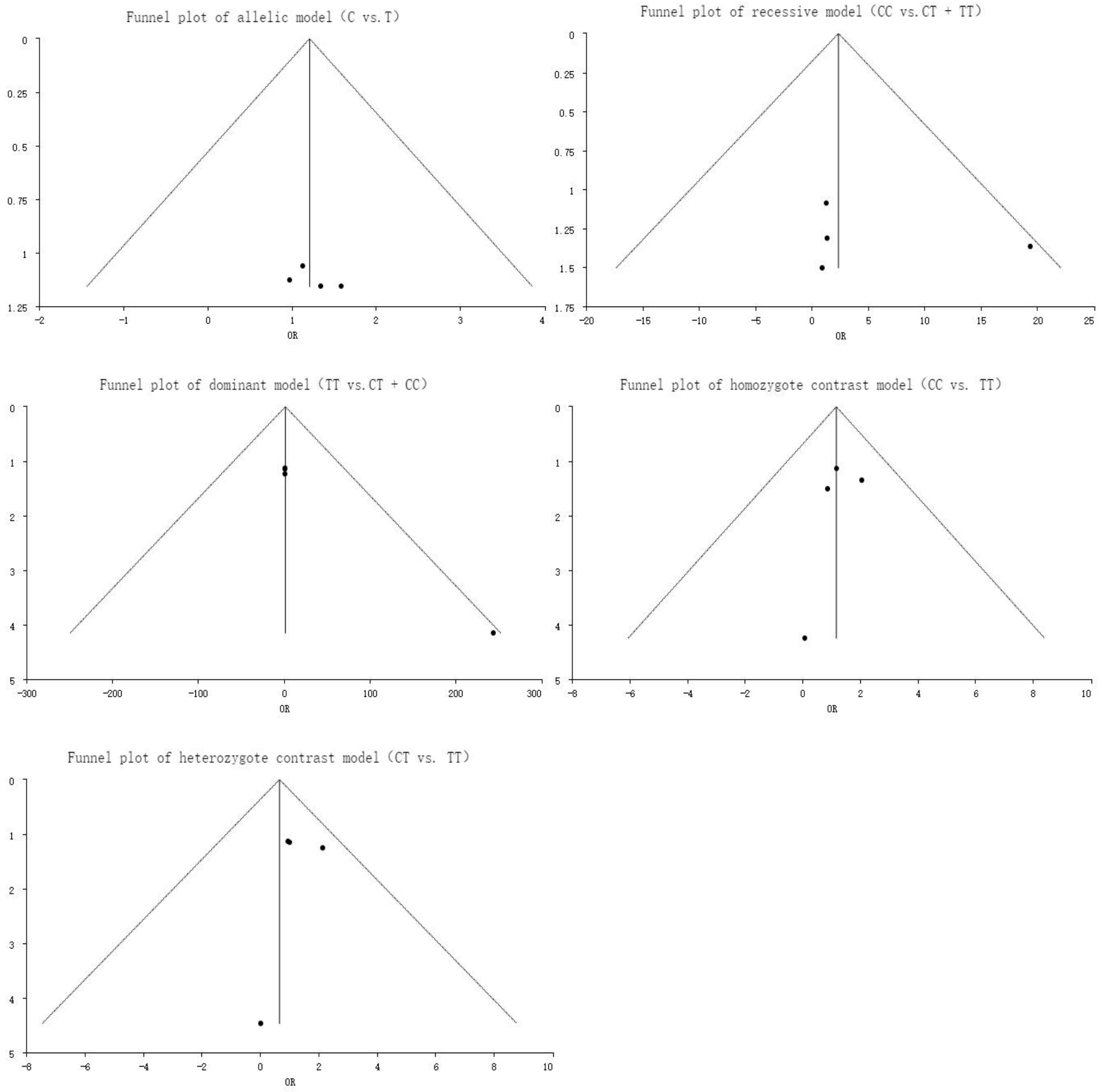

Figure S1 Funnel plots of TLR1 rs4833095

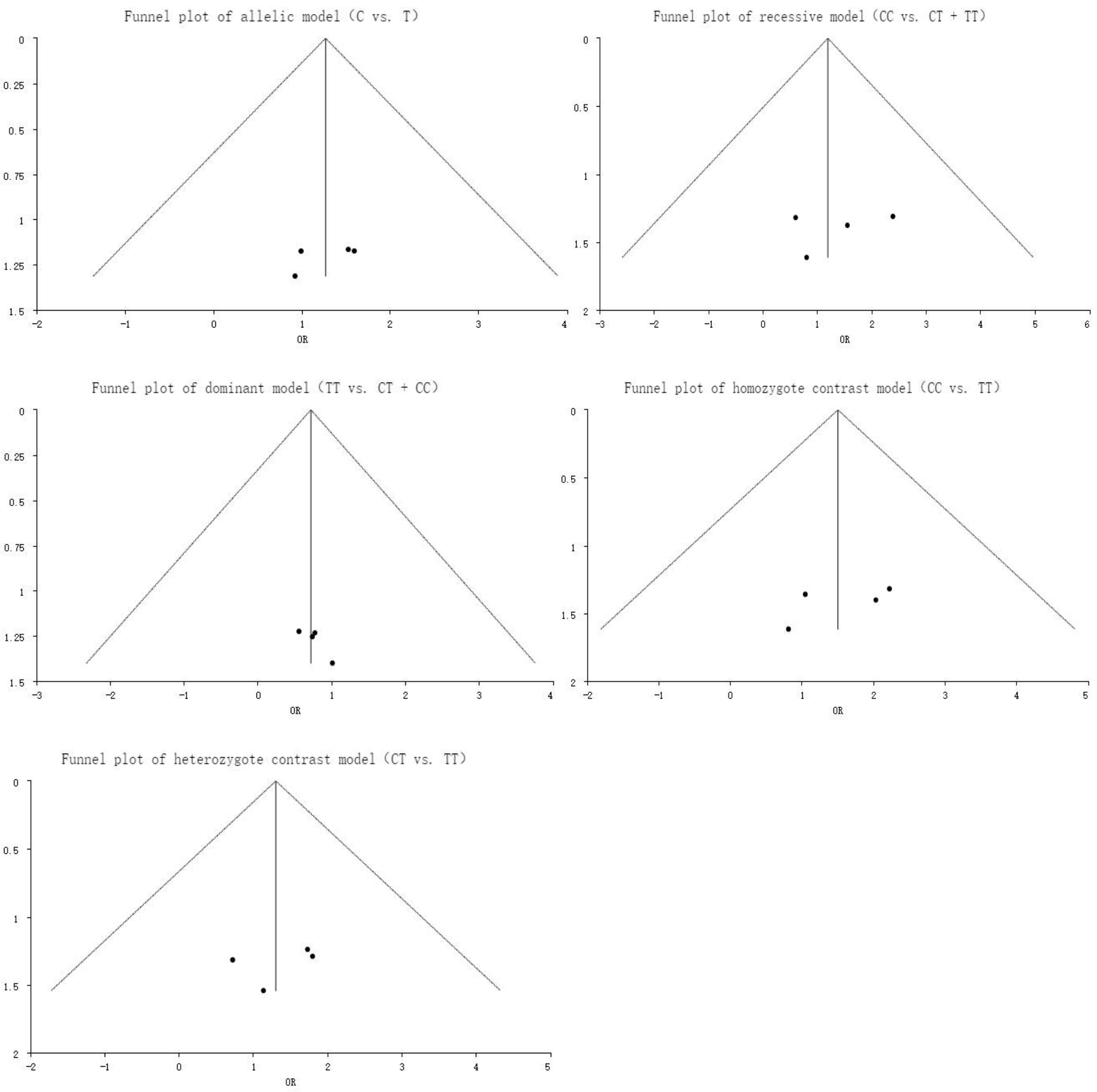

Figure S2 Funnel plots of TLR2 rs3804099

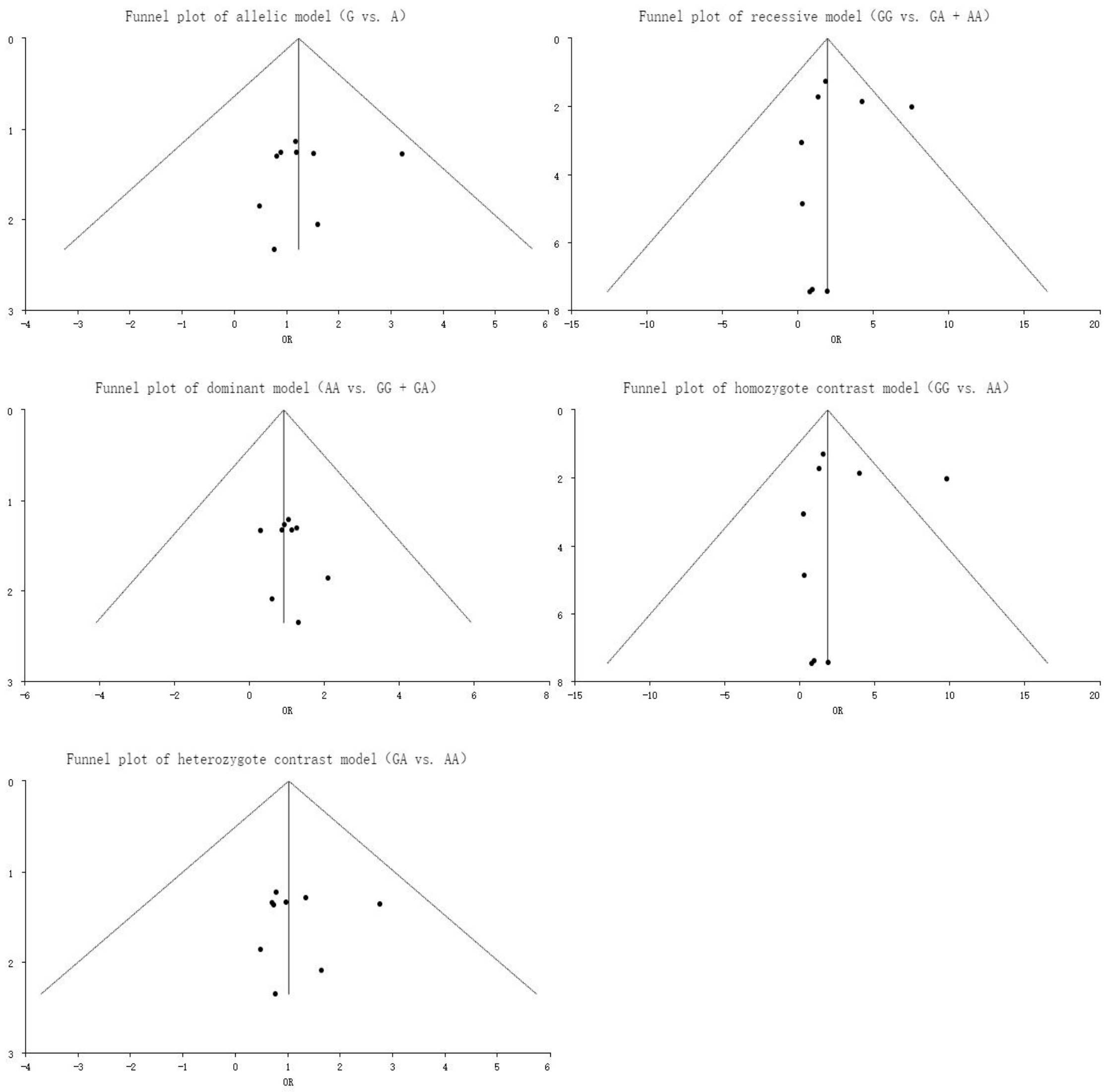

Figure S3 Funnel plots of TLR4 rs4986790

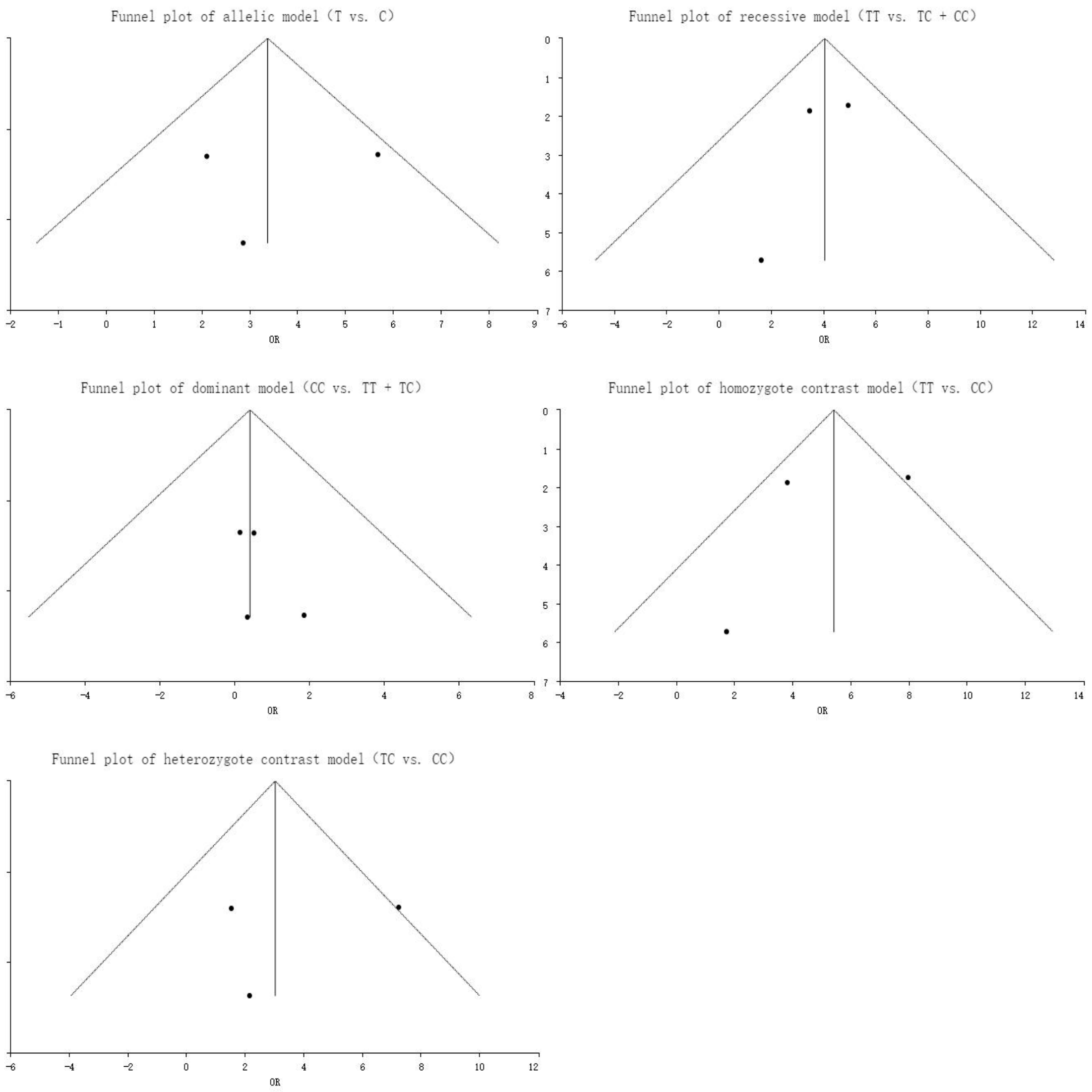

Figure S4 Funnel plots of TLR4 rs4986791

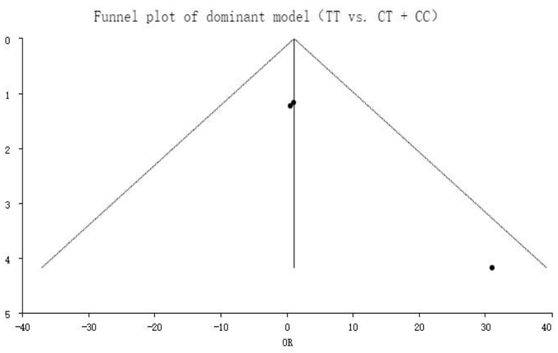

Figure S5 Funnel plots of TLR5 rs5744174

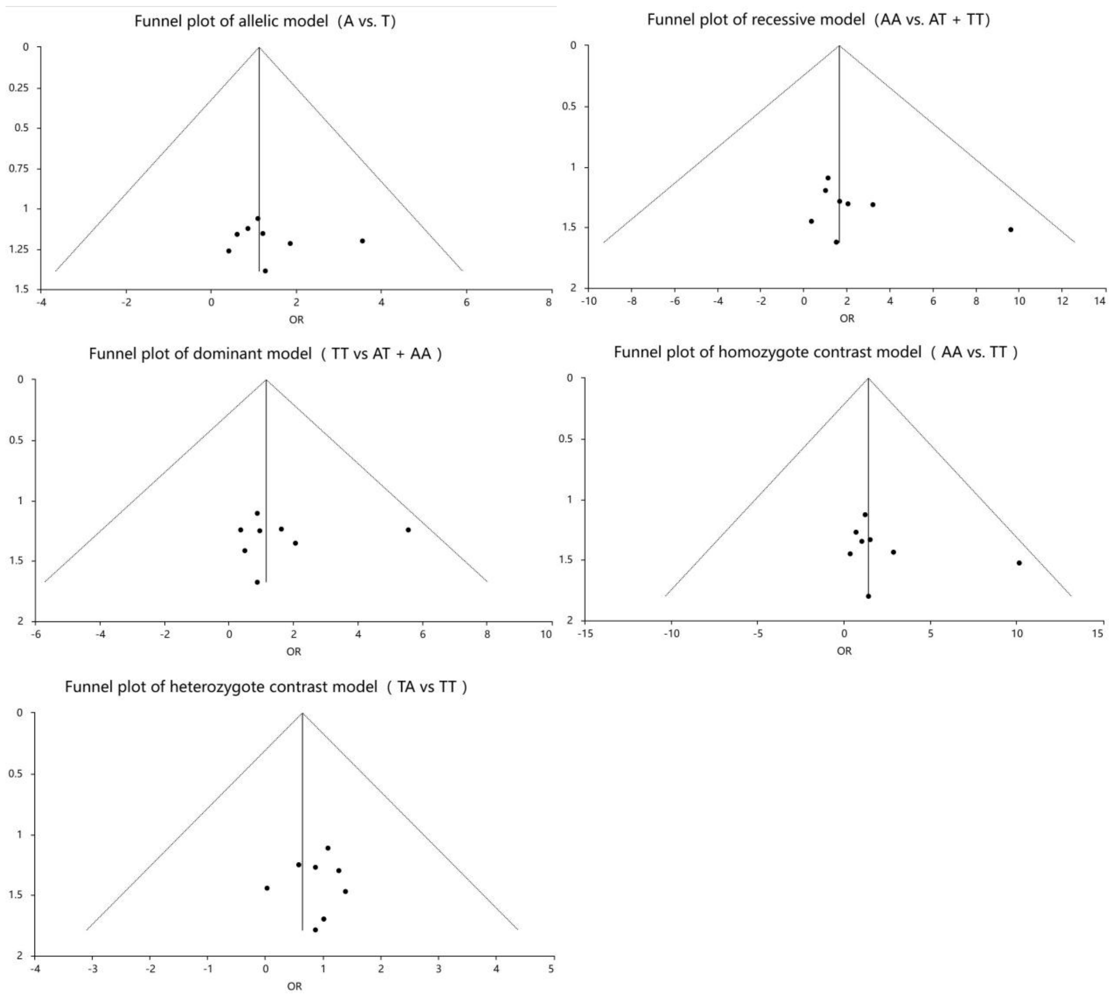

Figure S6 Funnel plots of TLR10 rs10004195
